# Supplementary material for: Curated and harmonised transcriptomics datasets of interstitial lung diseases
Source: Data Brief. 2025 Oct 14;63:112139. doi: 10.1016/j.dib.2025.112139 (PMC12581653; doi:10.1016/j.dib.2025.112139)
Supplement: Supplementary file 1 [file mmc1.zip › Supplementary_material/RNA-seq/fastQC reports/GSE169500/SRR14057114_fastqc.html]

SRR14057114.fastq.gz FastQC Report 

FastQC Report

Mon 5 Feb 2024  
SRR14057114.fastq.gz

## Summary

- Basic Statistics
- Per base sequence quality
- Per sequence quality scores
- Per base sequence content
- Per sequence GC content
- Per base N content
- Sequence Length Distribution
- Sequence Duplication Levels
- Overrepresented sequences
- Adapter Content

## Basic Statistics

| Measure | Value |
| --- | --- |
| Filename | SRR14057114.fastq.gz |
| File type | Conventional base calls |
| Encoding | Sanger / Illumina 1.9 |
| Total Sequences | 16060745 |
| Sequences flagged as poor quality | 0 |
| Sequence length | 25-380 |
| %GC | 50 |

## Per base sequence quality

## Per sequence quality scores

## Per base sequence content

## Per sequence GC content

## Per base N content

## Sequence Length Distribution

## Sequence Duplication Levels

## Overrepresented sequences

| Sequence | Count | Percentage | Possible Source |
| --- | --- | --- | --- |
| ACACTGAGGACTCTGTTCCTCCCCTTTCCGCCTAGGGGAAAGTCCCCGGA | 295043 | 1.8370442965130198 | No Hit |
| GGGAATGTCTACGTGCGTATGCACGTGGCACTCTCTGCCCGAGGTCCGGG | 268750 | 1.6733345806810331 | No Hit |
| GGGCCACGAGCTGAGTGCGTCCTGTCACTCCACTCCCATGTCCCTTGGGA | 150818 | 0.9390473480526589 | No Hit |
| AAGCTCAGGGAGAGCCCTGTTAGGGCCGCCTCTGGCCCTAGTCTCAGACC | 139845 | 0.8707254862710292 | No Hit |
| GCTGCTACAGGAGAATAGCAGACAGGTATAGTTAAGAAACAACTTTATTT | 128242 | 0.7984810169142216 | No Hit |
| ACCAATGTCATAGGGTGCAATATCTACAATAGGTAGTCTCACAGCCTTGC | 124484 | 0.7750823514102241 | No Hit |
| TTTACACCTGCTAGGGTGTTCAAAGGTCAGTGCTATAGAAATTCAGTATC | 102984 | 0.6412155849557415 | No Hit |
| CTAAGTGCTGTGTTGTCGTTCCCCCTGCTTAAAATAAAGTTGTTTCTTAA | 100591 | 0.626315902531296 | No Hit |
| CAATATCAAGGAAGGGCAGGCGTGATGGCTTATTTGTTTTGTATTCAATG | 65866 | 0.41010550880423047 | No Hit |
| TGGTTGCACGAAACACACTGGGGAATGGAGCAAAACAGTCTTTGAATATC | 60745 | 0.3782203129431418 | No Hit |
| AAAGCCAAGAAAACCAACGATGCCAGATACTGAATTTCTATAGCACTGAC | 56823 | 0.35380052419735203 | No Hit |
| TTCTTGTAGATGGCTGCTCTAAAAAGACAAATGAATGGGGAAAGACAATC | 54628 | 0.3401336612965339 | No Hit |
| TCTGCAATGCAATCACAATGCCCAAACTAGACCTGCCATTTCTCACACAC | 51886 | 0.3230609788026645 | No Hit |
| AGAGCATGACCGATGGATTCCAGTTCGAGTATGGCGGCCAGGGCTCCGAC | 48017 | 0.2989711872020881 | No Hit |
| TGGCTCTCCCAGAGGCAGAAGGACAATGGCTGTTTCAGGAGCTCTGGGTC | 47880 | 0.2981181757135176 | No Hit |
| TCTGTCTTCTTCAGTTTCGACTTATCGAATTTCTCGATCTCAGCCATATC | 45107 | 0.2808524760215046 | No Hit |
| TGTTGTGCTAAACGCTGGGAGGGTACAAGTTGGTCATTCCTAAATCTGTG | 40197 | 0.25028104238003906 | No Hit |
| ATTCTTCCTACCCATGAGCATGGAATGTTCTTCCATTTGTTTGTATCCTC | 37660 | 0.23448476393841008 | No Hit |
| TTTTTGTTGCCTGTAAAGTGCTCTGGCATTGCCTGAGGATAGATGAGAAA | 37503 | 0.23350722522523085 | No Hit |
| GAGAGGGTCACTTCATCTTCTACTCCTCCCTTTATGGCATTGTTGAGCAG | 35882 | 0.22341429367068588 | No Hit |
| TGGTTCCTGGCAAGCCCATGTGTGTTGAGAGCTTCTCAGACTATCCACCT | 32782 | 0.20411257385631862 | No Hit |
| ATGTTCTGGGAGGCCTCGGTGGACATCAGGCGCAGGAAGGTCAGCTGGAT | 32256 | 0.20083750784910662 | No Hit |
| ACACCCACCGCAACTGTCTGTCTCATATCACGAACAGCAAAGCGACCCAA | 30555 | 0.19024646739612638 | No Hit |
| GGTTATAATCATTGGCAATGTTGTAATCAACAACCCATGTTATTTCTACA | 27650 | 0.17215888802169516 | No Hit |
| TGAATCTTGAACTGAGTTCCACTTGTAAACTTCTTGTTTCTTGTGGTTCC | 26478 | 0.16486159265961822 | No Hit |
| AGAGGCCACAGTAAGGCCCATCCGAGGTCCTGAGGAGATCTTTTCTCTCT | 26141 | 0.16276330892495958 | No Hit |
| AGCGGTTCCGCTGCCCTGAGGCACTCTTCCAGCCTTCCTTCCTGGGCATG | 25737 | 0.1602478589878614 | No Hit |
| ACACTGAGGACTCTGTTCCTCCCCTTTCCGCCTAGGGAAAGTCCCCGGAC | 25385 | 0.15805617983474615 | No Hit |
| CACTTGGCTTCACCCGTGTGACTTTCGTAACGGGGAGAGAGAGAAAAGAT | 25076 | 0.15613223421453987 | No Hit |
| TACTCGTGCGCCTCGCTTTGCTTTTCCTCCGCAACCATGTCTGACAAACC | 25045 | 0.15593921701639618 | No Hit |
| TCACACTTCATGATGGAGTTGAAGGTAGTTTCGTGGATGCCACAGGACTC | 25035 | 0.15587695340409177 | No Hit |
| GAAGACGTACCTTTGTTGGAAGACATACCTTTGTTGGAAGACGTACCTTT | 24454 | 0.1522594375292055 | No Hit |
| AAAACAGCGTCTTACTGGGGAGGGATATGTGCTTTCTCATCTATCCTCAG | 24193 | 0.15063435724806043 | No Hit |
| TGTTCCCTCTCCTCATGAGATTGGTGAAGAAAGTATTTGGCAAAGTTCTT | 23888 | 0.14873531707277587 | No Hit |
| TCTTTCTGGCCTGGAGGCTATCCAGCGTACTCCAAAGATTCAGGTTTACT | 22376 | 0.13932105889234903 | No Hit |
| TCTTCCAGCCTACTTGTGTCTTCCAACAAAGGTACGTCTTCCAACAAAGG | 22117 | 0.13770843133366478 | No Hit |
| AGTGTGGCGATTCCTCAGGGATCTAGAACTAGAAATACCATTGACCCAGC | 21785 | 0.13564127940515835 | No Hit |
| TGACTGGTGATGCTGATATTGGAGTGCATCTGAGGCTGTTGTAGAAATAA | 21255 | 0.1323413079530246 | No Hit |
| TCTTCAAGGAGAACTACAAACCACTGCTCAAGGAAATAAAAGAGGATACA | 20886 | 0.13004378065899186 | No Hit |
| CAGCATGATTTATAGTCCTTTGGGTATATACCCAGTAATGGGATGGCTGG | 19994 | 0.12448986644143843 | No Hit |
| TGCGAAAAGTTACTTTCAATGGACTGAACCAGATGATTGTCATAGAACTG | 19506 | 0.12145140216098319 | No Hit |
| TGTCCATGTCTTACTACTTTGACCGCGATGATGTGGCTTTGAAGAACTTT | 19338 | 0.1204053734742691 | No Hit |
| AACCCGCGTGCAACCTGTCCCGACTCTAGCCGCCTCTTCAGCTCGCCATG | 18067 | 0.11249166835037852 | No Hit |
| CAGAAGCTCTTTATGTCAAACTTCTCAAGTTGATTGACCTCCAGGTAATA | 18027 | 0.11224261390116089 | No Hit |
| TGCATATACTGGTTAGTGAGGTGAGCCTGGCGCTCTTCTTTGCGCTGAGC | 16978 | 0.10571116097042822 | No Hit |
| CAGGAAAGGCTTAAGAGAGATATCTCCGTCACAAGCCTTTGCCATCTCCG | 16663 | 0.10374985718283927 | No Hit |
| CCCTGTTCCTACTCTCTCACTCCAGTTACCACAATTTCTCTCCAGCAACT | 16087 | 0.10016347311410524 | No Hit |

## Adapter Content

Produced by FastQC (version 0.11.7)
